# DNA sequencing of anatomy lab cadavers to provide hands-on precision medicine introduction to medical students

**DOI:** 10.1186/s12909-020-02366-0

**Published:** 2020-11-16

**Authors:** Ramu Anandakrishnan, Tiffany L. Carpenetti, Peter Samuel, Breezy Wasko, Craig Johnson, Christy Smith, Jessica Kim, Pawel Michalak, Lin Kang, Nick Kinney, Arben Santo, John Anstrom, Harold R. Garner, Robin T. Varghese

**Affiliations:** 1 Edward Via College of Osteopathic Medicine, (VCOM), VA, Biomedical Sciences, 2265 Kraft Drive, Blacksburg, VA 24060 USA; 2 Gibbs Cancer Center and Research Institute, Spartanburg, SC 29303 USA; 3 Virginia Department of Health, Richmond, VA 23219 USA

**Keywords:** Precision medicine, DNA sequencing, Exome sequencing, Histology, Clinically informative variants, Anatomy lab

## Abstract

**Background:**

Medical treatment informed by Precision Medicine is becoming a standard practice for many diseases, and patients are curious about the consequences of genomic variants in their genome. However, most medical students’ understanding of Precision Medicine derives from classroom lectures. This format does little to foster an understanding for the potential and limitations of Precision Medicine. To close this gap, we implemented a hands-on Precision Medicine training program utilizing exome sequencing to prepare a clinical genetic report of cadavers studied in the anatomy lab. The program reinforces Precision Medicine related learning objectives for the Genetics curriculum.

**Methods:**

Pre-embalmed blood samples and embalmed tissue were obtained from cadavers (donors) used in the anatomy lab. DNA was isolated and sequenced and illustrative genetic reports provided to the students. The reports were used to facilitate discussion with students on the implications of pathogenic genomic variants and the potential correlation of these variants in each “donor” with any anatomical anomalies identified during cadaver dissection.

**Results:**

In 75% of cases, analysis of whole exome sequencing data identified a variant associated with increased risk for a disease/abnormal condition noted in the donor’s cause of death or in the students’ anatomical findings. This provided students with real-world examples of the potential relationship between genomic variants and disease risk. Our students also noted that diseases associated with 92% of the pathogenic variants identified were not related to the anatomical findings, demonstrating the limitations of Precision Medicine.

**Conclusion:**

With this study, we have established protocols and classroom procedures incorporating hands-on Precision Medicine training in the medical student curriculum and a template for other medical educators interested in enhancing their Precision Medicine training program. The program engaged students in discovering variants that were associated with the pathophysiology of the cadaver they were studying, which led to more exposure and understanding of the potential risks and benefits of genomic medicine.

**Supplementary Information:**

The online version contains supplementary material available at 10.1186/s12909-020-02366-0.

## Background

Since the publication of the human genome sequence in 2003, clinical medicine has undergone a revolution with genomic data informing treatment decisions and being used alongside phenotypic data to manage disease. This revolution can be attributed to decreased cost, increased efficiency, and faster output of DNA sequencing technologies [1]. The success of large-scale genome studies and tailored cancer treatments customized for subsets of patients with specific genotypes has driven the incorporation of genomics into the practice of medicine. There are currently more than 3000 genes known to cause inherited genetic diseases and many more associated with complex diseases and drug responses [2]. The increased availability of individual genome and exome sequencing has the potential to improve treatment through targeted personalized medicine.

Precision Medicine takes into account genetic variability between individuals which can be applied not only during diagnosis, treatment, and management of disease, but also through appropriate screening for disease markers in individuals with a family history of particular diseases or conditions. The primary way that Precision Medicine has been applied to date has been through companion diagnostic tests for an identified variant that indicates whether a candidate pharmaceutical will work effectively for a patient with that variant, and with minimal or known side effects [3]. Another way Precision Medicine has been implemented is through tests for specific disease susceptibilities. Lastly, Precision Medicine has also included single patient genome sequencing to identify the root cause of a particular disease or trait, often in the case of a suspected novel monogenic disease [4]. These applications of Precision Medicine, together with the continually decreasing cost of genome sequencing, suggest that genome sequencing for a majority of patients will become standard practice in the near future [5–8].

As more patients research diseases on the internet and use commercially available genetic tests such as 23andme, physicians, especially primary care clinicians, will be called upon to be knowledgeable of the field of Precision Medicine and be able to advise their patients. Are physicians currently trained to handle the onslaught of questions patients will be asking them regarding genetic tests? Currently most medical students only receive classroom lectures on Precision Medicine, which does not provide a realistic understanding of its potential and limitations. The few clear examples typically used in classroom lectures designed to address learning objectives such as “Identify examples of how genetic variation can predict disease risk and guide treatment strategies”, fail to address the diversity and ambiguity found in real-world genotyping. To close this gap, we have implemented a hands-on Precision Medicine program utilizing exome sequencing to prepare a report resembling a clinical genetic report of cadavers studied in the anatomy lab. We have extended previous attempts to implement Precision Medicine [9] in three ways. 1. We utilized blood and tissue samples from cadavers in the anatomy lab and performed exome sequencing to produce clinically relevant genomic reports representing what students will see in their clinical experience. 2. We incorporated descriptions of the research tools used in genome analysis into lectures and appended an additional lecture in the anatomy lab introducing software to further investigate genomic variants. 3. We utilized an interdisciplinary approach involving histology, anatomy, and Genetics to better understand how genomic variants found in a “patient” could influence health conditions that were apparent during dissection. We used three sample sets comprised of pre-embalming blood samples and tissue samples. DNA from these samples was sequenced and analyzed to produce genomic reports identifying clinically significant gene variants. These reports were compared to dissection notes and correlations were noted either by faculty or by students. Some variants identified had potentially significant implications for disease management. On the other hand, many of the variants did not correlate to cause of death or anatomical findings, demonstrating the importance of treating genomic reports with caution. These findings reinforced the classroom learning objective “Identify examples of how genetic variation can predict disease risk and guide treatment strategies” through real-world examples. Overall, the approach presented here offers a practical way to introduce students to Precision Medicine and help future physicians to answer patient questions about clinical genomic reports.

## Methods

**Fig. 1:**
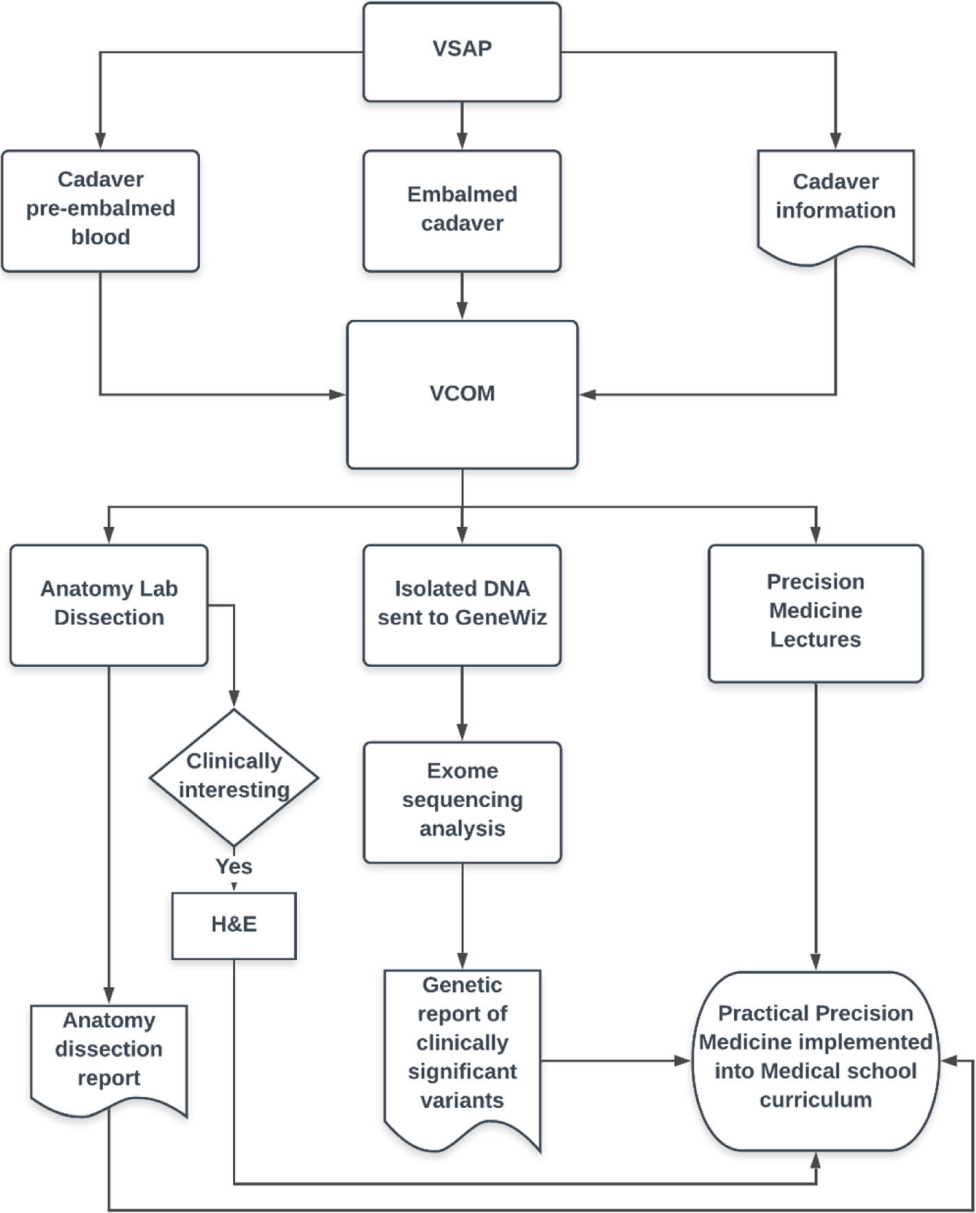
Hands-on Precision Medicine training process. DNA was isolated from pre-embalmed blood and from embalmed tissue samples, from cadavers provided by the Virginia State Anatomical Program (VSAP). Genetic reports were prepared from exome sequence analysis. H&E staining was performed for a pancreatic cancer case. Clinically relevant variants noted in the genetic report were compared to cause of death and anatomical findings noted in the dissection report, and discussed with the students

We used DNA sequencing data for anatomy lab cadavers to provide students with a more practical exposure to Precision Medicine. Figure 1 illustrates the overall process used for the hands-on Precision Medicine training using DNA sequencing of blood and tissue samples. Described below are details of how a practical introduction to Precision Medicine was integrated into the anatomy lab along with details of the processes for blood and tissue sampling, DNA isolation, sequencing, and data analysis. In addition to analyzing the relevance of genetic findings to patient care, four students who expressed an interest in participating in research, were also engaged to perform tissue sample collection, DNA isolation, analysis of sequencing data and preparation of genetic reports under the guidance of faculty. Of importance, all cadavers were donated for science and teaching and de-identified before we received or processed any samples. We did/will not attempt to re-identify donors from sequencing data, which will provide additional protections for the donors and their relatives and associates. We report only high-level findings (without enough sequence information for others to re-identify donors). This project was approved by the Institutional Review Board (IRB).

### Leveraging the anatomy lab to introduce students to precision medicine

Our current curriculum includes a course on Genetics during Block 1 of our Doctor of Osteopathy program. Each block is approximately two and a half months long. The course includes four lectures that relate to Precision Medicine, including Pharmacogenetics/Pharmacogenomics and Personalized Medicine, Cancer Genetics I and II, and Genetic Counseling. These lectures cover the basics of therapeutic strategies based on individual genetic traits. However, the simplistic examples in these lectures, while highlighting the potential of Precision Medicine, fail to drive home its complexities, ambiguities and limitations. A more realistic understanding would require students to decipher the potential relevance of a representative genetic report for an individual’s medical condition and treatment. We attempted to address this need by leveraging student dissections in the anatomy lab, given that some pathologies visible during dissection would correlate with known genetic markers.

The anatomy course spans Block 1 through Block 5, with a combination of lectures and laboratory sessions. Our anatomy lab has 32 cadaver tables with groups of 5–6 students assigned to each table and additional tables for faculty prosections. A lecture was added to the start of Block 5 supporting Genetics learning objectives including, (1) students will identify tools used in investigating clinically relevant genomic variants and (2) students will identify the evolving nature of genomic medicine including pitfalls of Precision Medicine. The lecture was designed to introduce students to the concept of Precision Medicine, describe software used to search for clinically relevant genetic variants and associated diseases, explain how to read a genetic report, and expand on the details of their findings. Before the lecture, students working with donors that had been sequenced were provided with a copy of the genetic report (described below) for their cadaver. These reports were distributed at the beginning of Block 5 due to the time required to extract and sequence DNA and prepare the genomic reports. The students were instructed to look up pathogenic variants identified in the report on online databases, such as SNPedia and ClinVar, consider the variants in relation to their cadaver based on observations and their dissection reports from Blocks 1–4, and during Block 5, keep in mind findings contained in their genetic report while working on their cadaver. Students are required to maintain detailed cadaver dissection reports throughout each block, and were thus able to compare notes from previous dissections to the genetic reports for their donors. These notes included any anatomical variants or anomalies, any possible pathology, and evidence of surgical procedures. Laboratory instructors include a pathologist and retired surgeon, an osteopathic physician, and Ph.D. trained scientists. Visible pathologies and variants were discussed as the students work through each dissection. Students at tables without genetic reports were encouraged to collaborate with those who had them, and the donors who were sequenced were distributed around the lab in such a way as to make them accessible to all students. During lab sessions, students discussed specific findings in the genetic report that might be relevant to their cadaver’s medical condition and could have factored into treatment. The students also noted in their dissection reports whether anomalies or pathologies correlated with their genetic reports. It was these correlations that seemed to be most valuable in the estimation of the students, based on their feedback after the course. From the true positives, false positives and false negatives in the report, the students were able to observe first-hand the potential and challenges associated with the practical application of Precision Medicine.

### Sample collection and DNA isolation from blood

This project was reviewed and received IRB approval (IRBNET ID: 1129894–1 & 1,308,833–3). Blood was collected from twelve donated cadavers (donors) for serological testing prior to embalming by the Virginia State Anatomical Program (VSAP), a department of the Virginia Department of Health and a willed donor program. Donors were gifted for medical research and accepted to VSAP based on three criteria: authorization or consent by legal next of kin, acceptable condition of donor after death, and proper reporting of death. Donor information provided by VSAP is shown in Table 1. A total of 4 ml of blood was drawn into a BD Vacutainer blood collection tube with K2EDTA. Samples were then stored at refrigeration temperatures until they were shipped to our lab in an insulated container with freezer packs. Upon arrival, DNA was isolated using the Qiagen DNeasy Blood and TissueKit.

**Table 1:** Donor information for cadaver (donor) samples. Information provided by Virginia State Anatomical Program for the fourteen cadavers used in this study. Blood samples were collected from twelve of the samples. Tissue samples were collected from donor #292 to determine if sufficient DNA could be isolated from embalmed tissue, and from donor #250 for a more detailed case study of pancreatic cancer

Donor #	Sample	Cause of Death (COD)	Other health information
272	Blood	Alzheimer’s dementia	
275	Blood	Multiple myeloma	
280	Blood	Colon cancer	
281	Blood	Alzheimer’s dementia	
284	Blood	Metastatic lung cancer	
286	Blood	Cardiogenic shock, cardiac arrest	Severe ischemic cardiomyopathy, CAD, acute hypoxic respiratory failure
293	Blood	CVA with late effect	
298	Blood	Ovarian cancer	
303	Blood	Aspiration pneumonia with hypoxia	Erosive esophagitis/GERD
306	Blood	Acute CVA	
311	Blood	Cardiac arrest secondary to CAD	AFIB, Vascular dementia, H/O stroke, Seizure disorder
312	Blood	Metastatic squamous cell lung cancer	
250	Tissue	Metastatic pancreatic cancer	
292	Tissue	Aspiration pneumonia	AFIB, CHF, Dementia, Dysphagia

### Sample collection and DNA isolation from tissue

Tissue samples were collected from two embalmed cadavers received from the VSAP. Table 1 provides the available donor information for these two cadavers. Samples included hair, skin, skeletal muscle, liver, nerve, spinal cord and pancreatic tissue. Considerations for tissue collection included, first, that tissue isolation did not interfere with student anatomical dissection, and second that pure DNA samples were efficiently isolated in previous experiments as determined by nanodrop readings and gel electrophoresis analysis. DNA was isolated from samples using the Qiagen QiaAMP DNA FFPE Tissue Kit, excluding xylene treatment.

### DNA library preparation and HiSeq sequencing

Initial DNA sample quality assessment, DNA library preparation, and sequencing were conducted at GENEWIZ, Inc. (South Plainfield, NJ, USA). Sequencing results are summarized in Table S2 and details of the sequencing process are included in the Supplementary Information document.

### Analysis of whole exome sequencing data and variant annotation

Variant annotation and analysis consisted of five steps using a custom pipeline based on the GATK Germline short variant (Single nucleotide polymorphisms (SNPs) and Insertions/Deletions) discovery best practices [10]. (1) Sequencing data (reads), for blood and tissue samples, in FASTQ format were mapped to the human reference genome hg38/GRCh38 using the Burrows-Wheeler Aligner [11]. (2) Duplicate reads were removed using Picard’s MarkDuplicates function [12]. (3) Somatic variants were called utilizing GATK’s HaplotypeCaller function yielding a VCF file [13]. (4) The effect of these variants were determined using Variant Effect Predictor (VEP) [14]. (5) The annotated VEP file was then parsed using a custom Python script to separate variants based on clinical significance and to annotate with phenotype from the ClinVar database [15]. For gene ontology and pathway analysis we used the STRING [16] and Reactome databases [17]. For functional annotation of variants we used SNPnexus [18].

### Hematoxylin and eosin (H&E) staining

H&E staining was performed at LewisGale Hospital, Blacksburg, VA. Paraffin sections from the pancreas and liver tissues were deparaffinized by treatment with xylenes, alcohols and water. Following deparaffinization, the sections were stained in hematoxylin solution (10 min), rinsed in running tap water for 20 min, immersed in lithium carbonate for 3 s, rinsed in running tap water for 5 min and counterstained in eosin solution for 15 s. Following eosin staining, slides were dehydrated through two changes of 95% ethanol and two changes of 100% ethanol for 3 min each, cleared in two change of xylene for 5 min each, and coversliped using Permount.

## Results

A lecture on Precision Medicine was added to the anatomy lab, with the goal of providing students with a more practical perspective on the subject. This lecture and subsequent lab activities included a discussion of genetic variants identified through DNA analysis of cadaver blood and tissue samples. These variants ranged from protective to pathogenic. Pathogenic variants were compared to student dissection notes and possible implications discussed with the students. We were also able to confirm that sufficient DNA could be isolated from six of eight embalmed tissue types sampled (Table S1 and Figure S1). Included below is a more in depth genomic study of a pancreatic cancer case that had metastasized to the liver.

### Correlation between blood sample variants and cause of death (COD) or anatomical findings

**Fig. 2:**
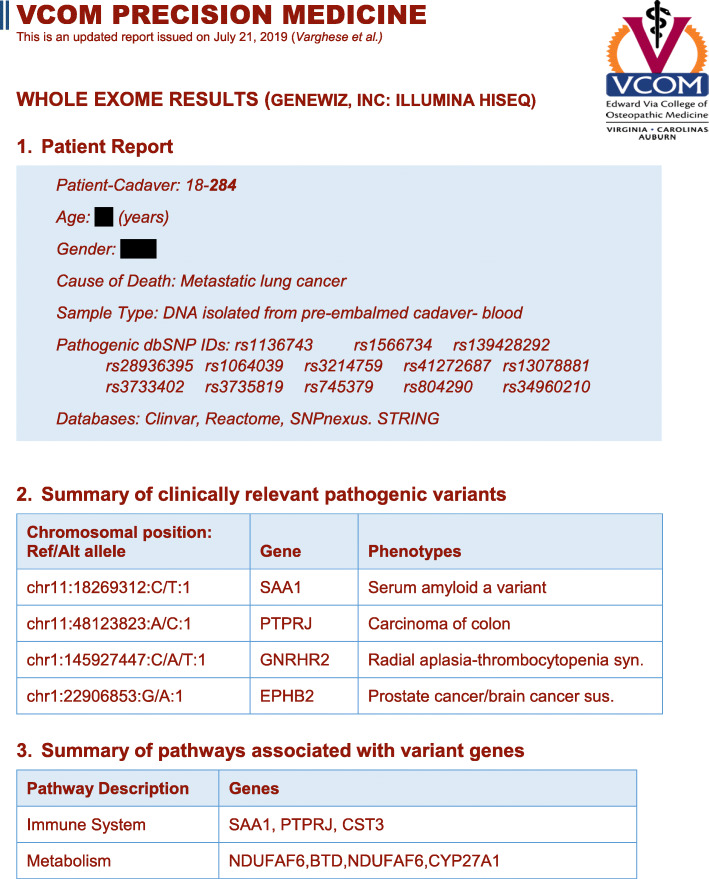
Illustrative genomic report. Report lists pathogenic variants identified from whole exome sequencing, and the pathways affected by the associated genes. The first page of one genomic report is shown here with the contents of all genomic reports summarized in Table S3

DNA from the twelve blood samples listed in Table 1 were sequenced and analyzed. Gel electrophoresis and quality control reports for whole exome sequencing are included in Supplementary Information (SI Fig. S1 and Tables S1/S2). Illustrative genetic reports were prepared for each sample identifying clinically relevant pathogenic variants and listing associated phenotypes. Figure 2 shows an example of one such report, with the data from all twelve reports summarized in Table S3. The students compared the clinical phenotypes associated with these variants to COD and anatomical anomalies identified and documented in their dissection notes. Students and faculty discussed the clinical significance of correlated findings during anatomy laboratory activities. Table 2 lists pathogenic variant phenotypes that correlated with cause of death or anatomical findings for all of the included donors. All but two donors (Donor 293 and 306) had pathogenic variants that may have been associated with increased risk for conditions or diseases noted in the cause of death and/or anatomical findings. In discussions with students, we noted that this does not necessarily imply a causal link. We also mentioned that, it may suggest a contributing factor that, with sufficient clinical evidence, may be taken into consideration when treating the patient. Following is a brief description of five cases of correlated variants and two cases of uncorrelated variants, and associated implications, discussed with students.

**Table 2:** Genetic variant phenotypes that correlated with cause of death or anatomical findings. Only correlated clinically relevant pathogenic variants are listed here. All pathogenic variants are listed in Table S3. Blank cells indicate that no correlated COD/anatomical findings were identified for the variant. ^a^ Student dissection reports for these donors were not available. (Gene names for each variant are found in genetic report (Fig. 2))

Donor #	Genetic variant Phenotypes	Correlated COD	Correlated anatomical findings
272	rs1136743: Serum amyloid A variant	Alzheimer’s dementia	Atrophy in medial temporal lobes – Alzheimer’s Disease
275	rs351855: Cancer progression and tumor cell motility	Multiple myeloma	
	rs3735819: Congenital heart disease		Larger than normal heart (cardiomegaly). Left side dominant and PFO.
280	rs6446482: Diabetes mellitus, noninsulin-dependent, association with	Colon cancer	
281	rs3735819: Congenital heart disease	Heart pathological findings suggest it may be related to cause of death.	Heart was vastly enlarged, and very thick hypertrophied ventricles. Stitches and scars along heart atria suggested surgery and aortic valve replacement. Vertebral artery on the left was abnormally large and the paired artery on the right was small, seemed to disappear around the cervical area
284	rs1566734: Carcinoma of colon	Metastatic lung cancer	Multiple scars and adhesions within the abdomen indicate multiple surgeries, this could be indicative of resections of tumors that eventually metastasized to the lungs as the total blood volume of the body must enter the lungs.
286	rs3735819: Congenital heart disease	Cardiogenic shock, cardiac arrest	• Sutures on aortic arch in two places—saphenous vein graft ×2? • Large heart, tearing of muscle fibers seen on outside • Position of grafts indicate ischemia of Rt ventricle • Heart 75% larger than normal • Pt does not have rt. auricle • Circumflex branches at root of aorta • Cusps for both aortic and pulmonary trunks arranged backwards
293	No variants with correlated COD or anatomic findings		
298^a^	rs10509305: Preeclampsia/eclampsia 4	Ovarian cancer	
303^a^	No variants with correlated COD or anatomical findings		
306	No variants with correlated COD/findings		
311	rs3735819: Congenital heart disease	Cardiac arrest secondary to CAD	Our cadaver’s cause of death was vascular related, so the IVC clamp, which was initially placed to prevent clots, supports vascular etiology of her death.
312	rs351855: Cancer progression and tumor cell motility	Squamous cell lung cancer	

### Cases with correlated variants

#### Donor 275

The cause of death for donor 275 was listed as multiple myeloma. Exome sequencing identified a pathogenic mutation in fibroblast growth factor receptors (FGFR4, rs351855) (Table 2). FGFRs play a critical role in cancer development through their angiogenic potential and enhancement of tumor growth. The rs351855 mutation has been associated with early lymph node and advanced tumor node metastasis [19, 20]. Students did not comment on gross findings that would correlate with this gene mutation, however, multiple myeloma can have various presentations and may not be obvious during dissection. Available diagnostics and treatments for FGFR were discussed with students, including FDA approved FGFR inhibitors, such as erdafitnib [21].

#### Donor 312

The cause of death for donor 312 was listed as squamous cell lung cancer. Exome sequencing revealed the same pathogen mutation in FGFR4 as in donor 275. Students noted a mass on the left third rib of unknown pathology, and a difference in texture between right and left lungs. SCLC typically originates in the parenchyma of the lung and may not have been located by the students. The same diagnostics and treatments discussed for donor 275 were discussed with students.

#### Donor 272

The cause of death for donor 272 was listed as Alzheimer’s dementia. Exome sequencing identified a pathogenic mutation in serum amyloid A (SAA, Table 2), specifically rs1136743. SAA is an apolipoprotein that is upregulated by inflammation and may contribute to amyloidosis, such as in Alzheimer’s disease [22, 23]. During dissection, students noted atrophy in medial temporal lobes, consistent with Alzheimer’s disease. Disease progression, management, and treatment options were discussed with students, along with the benefits of early detection.

#### Donor 284

The cause of death for donor 284 was listed as metastatic lung cancer. Exome sequencing identified a pathogenic mutation in protein tyrosine phosphatase receptor type J (PTPRJ), specifically rs1566734. PTPRJ is a tumor suppressor [24] and the rs1566734 mutation in PTPRJ has been linked to lung, colon and breast cancer [25]. Students made multiple observations consistent with this mutation, including abnormal tissue in the left lung, scars and adhesions on and in the abdomen consistent with multiple surgeries. Students proposed that the cancer may have originated in the abdomen and spread to the lungs, however, it would be impossible to know without access to past medical records. The utility of genetic testing when formulating a treatment plan for various types of cancer was discussed with the students.

#### Donor 295

The cause of death for donor 295 was listed as ovarian cancer. Exome sequencing revealed a pathogenic mutation in the DNA binding protein Storkhead Box 1 (STOX1), specifically rs10509305. In clinical studies, this mutation has been associated with preeclampisa [23], which has been estimated to more than double the risk of ovarian cancer. Students did not report any significant findings during dissection to correlate with the genomic findings, other than evidence of a unilateral mastectomy. The available genetic tests for preeclampsia and the consequences of untreated preeclampsia were explored with the students.

### Cases with uncorrelated variants

#### Multiple donors

The genetic reports for ten of twelve donors identified a pathogenic intron variant in GATA4 (rs3735819, Table S3). Interestingly, this variant is only found in 13% of “healthy” individuals based on the 1000 genomes study, and is associated with congenital heart disease. This variant is identified as pathogenic in the NIH ClinVar database based on a review article by Su et al. [26]. However, the supporting evidence is based on correlation with no experimental evidence suggesting causation. Considering that this particular intron variant occurs in 83% of our samples it is more likely that the “correlated” heart disorders for four patients with this mutation (Table 2) are coincidental, even though the anatomical findings for donor 286 shows cusps for both aortic and pulmonary trunks arranged backwards, indicating a congenital heart defect.

#### Donor 281

Another example of the limitations of genomic reports in clinical practice includes donor 281, a male. The genetic report for this donor includes the rs10509305 variant in STOX1. As noted above for donor 295, this mutation predisposes him to preeclampsia/eclampsia. However, this is irrelevant because of his gender.

These last two examples demonstrate that the evidence supporting the information in genomic reports need to be examined closely before advising patients. These considerations and limitations were discussed at length with students during the process of working through dissections and considering abnormal findings discovered as they worked. This speaks directly to the learning objective “students will identify the evolving nature of genomic medicine, including pitfalls of Precision Medicine.”

Another key limitation of the genetic variants identified in this study, and discussed with the students, is that many are not clinically actionable. We encouraged students to investigate the discovered variants using ClinVar and other resources given during the ancillary Precision Medicine lecture. The students they determined that many variants lacked a known treatment. It is important to note that the number of pathogenic variants that we discovered ranged from seven to fourteen variants per donor (Table S3), whereas two or fewer of these variants correlated with cause of death or anatomical findings. Although ClinVar may indicate multiple classifications for each genomic variant, for the purpose of this study we only selected variants with pathogenic classification. In addition, when performing exome analysis, the variant call files used data from the ClinVar database which is updated monthly. ClinVar classifications may change depending on time of analysis. The relatively large number of “pathogenic” variants that do not correlate to COD or anatomical findings demonstrate the need for caution, and the importance of supporting clinical evidence, when interpreting the results from a genomic study. The increased risk due to these variants may be low, the risk may be limited to a population subgroup, or the stated evidence supporting the pathogenicity of the variant may need further validation. It is also important to note that depending on the minor allele frequency of variants in a population, which may change depending on the available population genomic data, the identified variant’s frequency may not be significant enough to account for disease association.

As part of the Precision Medicine training, we emphasized the importance of considering several different factors when incorporating information from genetic reports into a therapeutic strategy. We demonstrated the importance of examining the reliability of supporting evidence and discussed conducting secondary tests to confirm the risk indicated by the variants. Another important piece of information provided by genetic analysis is the efficacy and toxicity of treatments to patients with specific variants. A 1998 study estimated that adverse drug reactions resulted in an estimated 2.2 million serious cases and over 100,000 deaths in 1994 [27]. The study indicated that many of these deaths could have been avoided if the physician had taken into consideration the patient’s genetic drug response variants’ efficacy and toxicity profile.

### Sufficient DNA for sequencing can be extracted from most embalmed tissue types

**Fig. 3:**
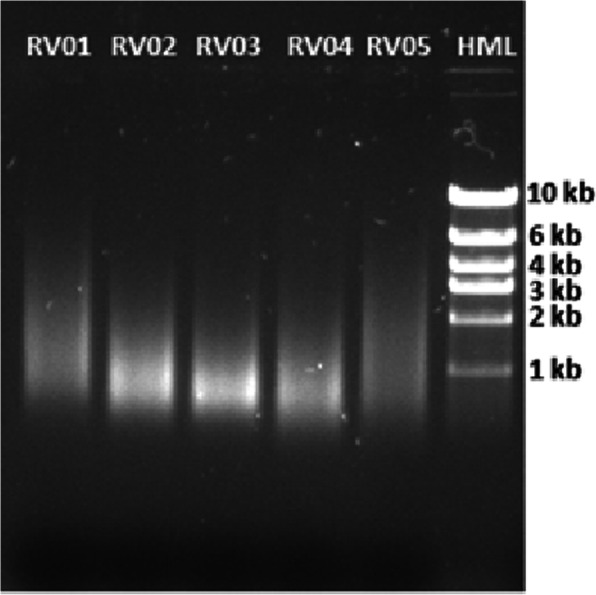
Gel electrophoresis results for tissue samples that were successfully sequenced. Samples RV01-RV05 correspond to sartorius muscle, striated muscle, gluteus maximus, liver and nerve tissue samples, respectively for donor 14

Genetic disorders, such as the pancreatic cancer case described below, can result from somatic mutations in addition to germline variants. To identify somatic mutations for a more detailed case study of such disorders, it is necessary to sequence DNA from relevant tissues. Extracting DNA from embalmed cadavers can be challenging [9, 28]. To confirm that sufficient DNA for sequencing can be obtained from embalmed tissue, we extracted DNA from eight different tissue types, including sartorius muscle, gluteus maximus, hair, liver, nerve, spinal cord and skin, all taken from Donor 292 (Table 1). Three medical students performed this work, with faculty supervision. All except for hair and skin yielded > 30 ng of DNA, sufficient for DNA sequencing (Fig. 3 and Table 3). Although the DNA was highly fragmented, it did not affect sequencing results. We obtained over 80 million reads for each sample submitted with a quality score > 93%. Subsequent experimentation demonstrated that sufficient DNA could be extracted from hair and skin samples as well with approximately three-fold higher yield by freezing the samples using liquid nitrogen, followed by the DNA extraction protocol described in Methods.

**Table 3:** DNA extraction and sequencing yield from different tissue types for Donor # 292. Yield and quality of DNA from sartorius muscle, striated muscle, gluteus maximus, liver, nerve and spinal cord tissue samples as measured measured by nanodrop and Qubit were sufficient for DNA sequencing, but not for hair and skin

Tissue Sample	Nanodrop A260/A280	Nanodrop A260/230	Qubit NA Yield (ng)	# Reads	Yield (Mbase)	Mean Quality Score	% Bases > 30
Sartorius Muscle	1.77	1.5	65.5	79,576,733	23,873	38.65	93.1
Striated Muscle	1.76	1.1	103	75,571,463	22,671	38.88	93.97
Gluteus Maximus	1.82	1.53	189	79,175,921	23,753	38.75	93.49
Hair	1.69	1.8	13.5				
Liver	1.87	2.22	600	68,240,142	20,472	38.8	93.66
Nerve	1.88	2	118	77,187,124	23,156	38.81	93.75
Spinal Cord Nerve	1.91	1.24	39.4	74,171,309	22,251	38.88	93.98
Skin	1.74	1.12	23.2				

Upon variant calling for these samples, we identified twenty clinically relevant pathogenic variants in the six samples (Table S3 B). One medical student performed this work, with faculty supervision. The number of such variants per sample varies from eight to eleven, with four of the variants occurring in all samples, and are likely germline variants. Thirteen variants occur in two or fewer samples. This mosaicism may arise from somatic variation or possibly from artifacts of embalming. For this reason, and because we can isolate DNA from blood at an earlier time-point, we will use pre-embalmed blood samples in our pipeline for the medical school curriculum, with tissue samples being used for more extensive case studies.

### Pancreatic cancer case with liver metastasis

Finally, to provide students a deeper understanding of the potential of Precision Medicine, we performed a case study of a donor whose cause of death was metastatic pancreatic cancer. Stage IV pancreatic cancer is currently an incurable disease. When diagnosed, it often has become locally advanced or even metastasized, and therapy is typically palliative. Therapy currently consists of combination chemotherapy, and the median survival is around six months following diagnosis.

**Fig. 4:**
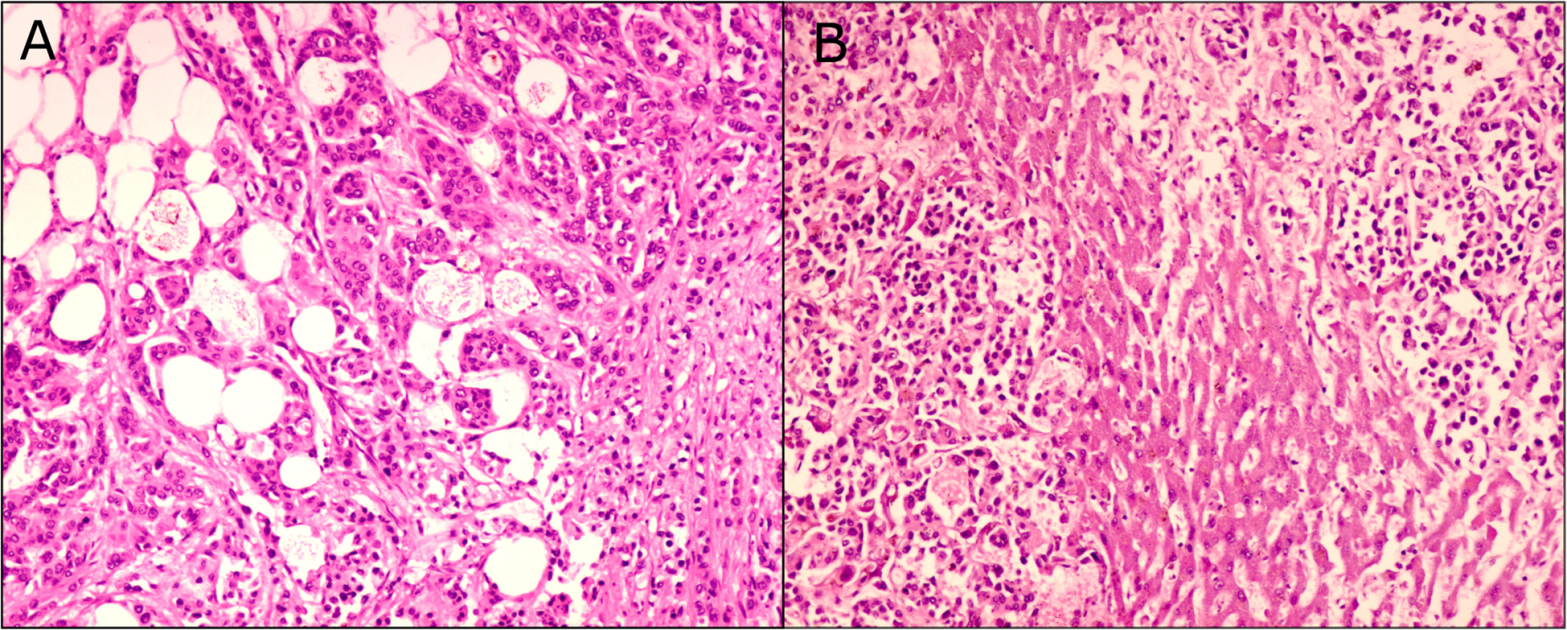
H&E staining of pancreatic and liver tumors. Left: Ductal adenocarcinoma of pancreas. Note infiltrating well-to-poorly formed ductal structures surrounded by remarkably desmoplastic stroma intermingled with adipocytes. Right: Histopathological features of liver metastasis. Note poorly formed ductal structures infiltrating the liver

**Fig. 5:**
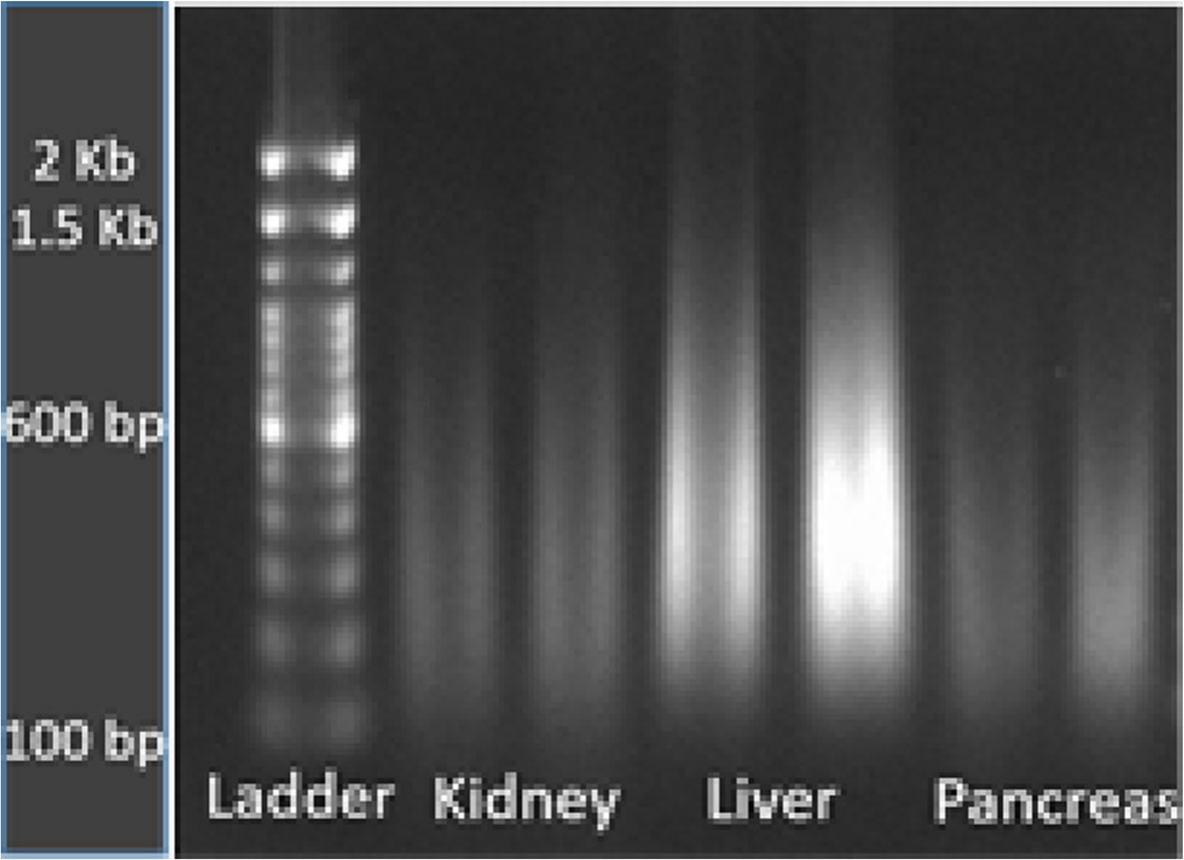
Gel electrophoresis of DNA extracted from primary pancreatic cancer lesion, and a metastatic lesion in the liver. DNA was successfully extracted from each respective tissue sample

The donor died from pancreatic cancer that had metastasized to the liver (Donor 250, Table 1). Tissue was collected from primary and metastatic tumors, and was processed for histological analysis. H&E staining and histological examination of pancreatic tissue samples suggested ductal adenocarcinoma (Fig. 4a). Liver metastasis was confirmed upon H&E staining and microscopic examination (Fig. 4b). Invasion of tumor cells into the normal architecture of the pancreas and liver are notable in stained tissues (Fig. 4). DNA was extracted from liver and pancreas and successfully sequenced (Fig. 5, Fig. S1 and Table S1). Analysis of sequencing data identified 11 pathogenic variants out of 200 total clinically relevant variants in the pancreatic sample and 41 pathogenic variants out of 300 clinically relevant variants in the liver sample (Table S4). Clinically relevant variants are categorized as pathogenic, likely pathogenic, confer a genetic risk, offer a protective factor, or affect drug response.

Breast cancer type 1 susceptibility protein (BRCA1) and Breast cancer type 2 susceptibility protein (BRCA2) mutations increase an individual’s risk of several cancer types, including pancreatic cancer. The protein truncating mutations rs397508986 in BRCA1 and rs80358998 in BRCA2, found in the liver and pancreas samples, are components of genetic screening protocols for breast and ovarian cancer [19]. Although these mutations are primarily associated with breast and ovarian cancer, protein-truncating mutations in these genes have been linked to pancreatic cancer as well [20–24]. It is plausible that these mutations contributed to the genesis of pancreatic cancer in this donor. Metastatic tumor samples from the liver contained 30 additional pathogenic mutations, including additional mutations in BRCA1 and BRCA2 (Table S4 C). These additional mutations are likely a result of defective BRCA DNA repair genes, and some of these mutations may have contributed to metastasis to the liver. Lastly, we discovered genetic variants that were associated with the efficacy and toxicity of chemotherapeutics. For example, rs2232228 affects the toxicity of anthracyclines [25], rs25487 affects the efficacy and toxicity of cisplatin and carboplatin [29], and rs3212986 affect the toxicity of cisplatin [30]. These chemotherapeutic and other platinum compounds are used in adjuvant and neoadjuvant chemotherapy as well as in advanced pancreatic cancer.

Following tissue collection and processing, students researched the disease etiology and pathology, and examined the slides along with a pathologist. They were directed to research the standard treatment for this type of cancer, and to consider the changes in gene expression between primary and metastatic tumors. Particular emphasis was placed on how a case such as this might be best managed, and how Precision Medicine can contribute to a well-informed and multifaceted treatment plan. Ultimately, the data was presented in poster format during a professional meeting.

## Discussion

Overall, the practical introduction to Precision medicine in the anatomy lab was positively received by students and faculty. The learning outcomes were evaluated using informal questioning during the end-of-the-course Anatomy evaluation. The survey indicated that the students appreciated the additional information and believed it would benefit them as practicing physicians. The end-of-year anatomy evaluation indicated that students understood how clinical genetic reports are compiled and they understood the risks as well as benefit of clinical genomic studies. We are now working with two of our other campuses to incorporate this training in their curriculum. The approach we utilize to prepare our students for Precision Medicine can be implemented in other medical schools with some important considerations: Institutional Review Board (IRB) approval, potential improvements based on our experience, cost of DNA sequencing and resources for data analysis. Each of these are discussed below.

Educators should work with their IRB contacts to obtain approval before proceeding with this program. The application for IRB review will need to detail steps that will be taken to ensure that the identify of donors will be protected. In addition, if a formal survey is to be conducted at the end of the program, separate IRB approval will be required to ensure that the survey is anonymous. The IRB will require details about the questionnaire, participant selection, data collection and analysis processes.

As we prepare to formally implement practical Precision Medicine training in our medical school curriculum and expand it to our other campuses, we have identified some potential improvement to the process. We plan to enhance the utility of the genetic report (Fig. 2) by replacing the chromosome position in section 2 of the report with the dbSNP ID from section 1 of the report. We will also consider adding a section listing drug response variants, which would be valuable in considering a treatment strategy. Section 3 of the genetic report identifies pathway affected by the genes with pathogenic variants. This information was not extensively discussed during this class, but can be valuable for interpretation of the variants with respect to biological processes and can aid in understanding possible mechanisms underlying the patient’s disease. In the next iteration of the training, we will expand on using the genetic report for this purpose and tie this information to elements of the biochemistry and cell biology curriculum. We also plan to enhance learning objectives to include Precision Medicine components, and to develop a more formal assessment for the students following the course.

The cost of DNA sequencing has significantly decreased but may still be too costly at approximately $400/sample. In addition, students, faculty or other support resources must be available who are familiar with basic biochemistry lab procedures such as DNA extraction, gel electrophoresis, and sample preparation. They must also be familiar with analyzing DNA sequencing data and the use of online bioinformatics tool. If not, bioinformatic analysis services are provided for a fee at many DNA sequencing cores. Finally, tissue processing and sequencing must be coordinated between schools and sequencing companies to get genomic reports to students’ before the conclusion of dissection activities. According to availability, blood or tissue can be used for sequencing, and students can volunteer for lab work or data analysis. Ideally, students would begin their dissection activities with the genetics report in hand, as the sequencing would have been performed using pre-embalming blood samples and the analysis complete before students began their dissection activities. Instructors would be aware of the variants present in the donors, or would at least have quick access to the information, so that they could be discussed as students identify any available evidence of phenotypes resulting from genetic variants. This real-time correlation between genotype and phenotype solidifies the utility of Precision Medicine in the mind of the student and can be extrapolated to future medical practice.

The educators involved in our project informed us that they were not burdened by any task in our approach and believed that students benefited from this training. This claim is supported by the new Precision Medicine case studies that were initiated as a result of findings from this project. Regarding educators concerned about the intricacies of genomic analysis or the costs associated with sequencing, an alternative approach is to sequence only a prosection cadaver and have the sequencing company perform the analysis. The company would send the educator a list of genetic variants that they could cross-reference in ClinVar and produce a similar clinical genetic report to complement their dissection and instruction.

## Conclusion

The achievement of sequencing the human genome has the potential to transform healthcare. With the rise of genetic testing and direct to consumer products, our physicians are encountering a new era in practicing medicine. To prepare our medical students for this transformation, we have established a protocol for incorporating practical Precision Medicine training into our medical curriculum. Our protocol entails DNA isolation from anatomy lab cadavers and exome sequencing of the DNA to produce genetic reports. These genetic reports resemble what our students will be exposed to in their clinical practice. Students experienced in the anatomy lab the potential benefits and limitations of Precision Medicine. An informal survey and end-of-class evaluation suggest that the hands-on Genetics program will better prepare our students to be physicians in the era of Precision Medicine. Students identified and discussed specific examples of cases where pathogenic variants correlated with cause of death or anatomical findings, illustrating how genetic markers with supporting clinical evidence may guide patient care. They also saw many more instances where pathological variants did not correlate with either, demonstrating the need for caution when interpreting the results of genomic reports. A pancreatic cancer case study combining histology, anatomy and genomics, demonstrated to the students how Precision Medicine can inform therapeutic strategy. The practical training presented here augments what is taught in the Genetics class, providing a more nuanced understanding of the limitations, potential and complexities of Precision Medicine.

## Supplementary Information

**Additional file 1:** **Table S1.** Quantitative analysis of DNA of blood and tissue samples using Qubit analyzer. **Figure S1.** Qualitative analysis of DNA from blood and tissue samples using gel electrophoresis. **Table S2.** Sample Sequencing Statistics for whole exome sequencing. **Table S3.** Pathogenic variants reported in genomic reports. **Table S4.** Overview of clinically relevant markers in pancreatic cancer and liver metastasis highlighting drug response and pathogenic variants.

## Data Availability

Modified de-identified whole exome sequencing data access can be provided as long as those requesting data agree not to attempt to re-identify cadaver or associate with any ancestry or other public or private forensics databases. Interested parties are encouraged to contact the corresponding author.

## Abbreviations

BRCA1: Breast cancer type 1 susceptibility protein
BRCA2: Breast cancer type 2 susceptibility protein
PCR: polymerase chain reaction
SNPs: Single nucleotide polymorphisms
VEP: Variant Effect Predictor
FGFR: Fibroblast growth factor receptors
STOX1: Storkhead Box 1
PTPRJ: Protein Tyrosine Phosphatase Receptor Type J
HE: Hematoxylin and Eosin
VSAP: Virginia State Anatomical Program
VCOM: Edward Via College of Osteopathic Medicine
IRB: Institutional Review Board
