# Supplementary material for: DNA sequencing of anatomy lab cadavers to provide hands-on precision medicine introduction to medical students
Source: BMC Med Educ. 2020 Nov 16;20:437. doi: 10.1186/s12909-020-02366-0 (PMC7670733; doi:10.1186/s12909-020-02366-0)
Supplement: Supplementary file 1 — Additional file 1: Table S1. Quantitative analysis of DNA of blood and tissue samples using Qubit analyzer. Figure S1. Qualitative analysis of DNA from blood and tissue samples using gel electrophoresis. Table S2. Sample Sequencing Statistics for whole exome sequencing. Table S3. Pathogenic variants reported in genomic reports. Table S4. Overview of clinically relevant markers in pancreatic cancer and liver metastasis highlighting drug response and pathogenic variants. [file 12909_2020_2366_MOESM1_ESM.docx]

**Supplementary Information for**

**DNA sequencing of anatomy lab cadavers to provide hands-on precision medicine introduction to medical students**

Anandakrishnan R^1,2,#^, Carpenetti T^1, #^, Samuel P^1^, Wasko B^1^, Johnson C^1^, Smith C^1^, Kim J^1^, Michalak P^1,^ , Kang L^1^, Kinney N^1,2^, Santo A^1^, Anstrom J^1^, Garner HR^1,2^, Varghese RT^1,2, *,#^

^1^ Edward Via College of Osteopathic Medicine, Biomedical Sciences, Blacksburg, VA 24060
^2^ Gibbs Cancer Center and Research Institute, Spartanburg, SC 29303
^#^ RA, TC and RV contributed equally to this study
^*^ Correspondence to [rvarghese@vcom.vt.edu](mailto:rvarghese@vcom.vt.edu)

**DNA library preparation and HiSeq sequencing**

Initial DNA sample quality assessment, DNA library preparation, and sequencing were conducted at GENEWIZ, Inc. Genomic DNA samples were quantified using a Qubit 2.0 Fluorometer (Life Technologies, Carlsbad, CA, USA) and DNA integrity was confirmed using 0.6% agarose gel with 50-60 ng samples loaded in each well. Samples were treated with NEB RNase I per manufacturer’s protocol, to remove single-stranded RNA. SureSelectXT Exome Enrichment System for Illumina Paired-End Multiplexed Sequencing Library and SureSelect Human All Exon V5 bait library were used for target enrichment DNA library preparation following the manufacturer’s recommendations and the standard low-input protocol (Agilent, Santa Clara, CA, USA), which starts with 200 ng. Briefly, the genomic DNA was fragmented by acoustic shearing with a Covaris S220 instrument. Fragmented DNA samples were cleaned up, end repaired, and adenylated at the 3’ends. Adapters were ligated to the DNA fragments, and adapter-ligated DNA fragments were enriched with limited cycle polymerase chain reaction (PCR). Adapter-ligated DNA fragments were validated using Agilent TapeStation (Agilent Technologies, Palo Alto, CA, USA), and quantified using Qubit 2.0 Fluorometer. A total of 750 ng of adapter-ligated DNA fragments were hybridized with biotinylated RNA baits at 65 °C for 24 hours. Hybrid DNA was captured using streptavidin-coated magnetic beads. Captured DNA was amplified and indexed with Illumina indexing primers. Post-captured DNA libraries were validated using Agilent TapeStation and quantified using Qubit 2.0 Fluorometer and quantitative PCR (Applied Biosystems, Carlsbad, CA, USA).

Illumina reagents and kits for DNA library sequencing cluster generation and sequencing were used for enrichment DNA sequencing. Post-captured DNA libraries were multiplexed in equal molar mass, and pooled DNA libraries were clustered on one lane of a flow cell, using the Illumina cBOT. After clustering, the flow cell was loaded on the Illumina HiSeq instrument according to manufacturer’s instructions. The samples were sequenced using a 2 x 150 paired-end configuration. Image analysis and base calling was conducted by the HiSeq Control Software (HCS 2.0) on the HiSeq instrument.

**Table S1. Quantitative analysis of DNA of blood and tissue samples using Qubit analyzer.** Qubit DNA quantification results for blood samples and tissue samples (donor #250) isolated using DNA isolated with Qiagen DNeasy Blood and TissueKit (Qiagen Catalog #69504).

| **Sample/Name** | **Sample Type** | **Sample Vol. (ul)** | **Qubit** | | | |
| --- | --- | --- | --- | --- | --- | --- |
|  |  |  | **Dilution Factor** | **Nucleic Acid Conc. (ng/ul)** | **Actual Nucleic Acid Conc. (ng/ul)** | **Total Amount (ng)** |
| Liver | genomic DNA | 62 | 1 | 28.20 | 28.20 | 1748.40 |
| Kidney | genomic DNA | 62 | 1 | 1.63 | 1.63 | 101.06 |
| Pancreas | genomic DNA | 62 | 1 | 1.16 | 1.16 | 71.92 |
| 281A | genomic DNA | 62 | 1 | 18.60 | 18.60 | 1153.20 |
| 311A | genomic DNA | 62 | 1 | 12.50 | 12.50 | 775.00 |
| 275A | genomic DNA | 62 | 1 | 9.50 | 9.50 | 589.00 |
| 284A | genomic DNA | 62 | 1 | 18.30 | 18.30 | 1134.60 |
| 272A | genomic DNA | 62 | 1 | 23.20 | 23.20 | 1438.40 |
| 298A | genomic DNA | 62 | 1 | 9.94 | 9.94 | 616.28 |
| 306A | genomic DNA | 62 | 1 | 29.00 | 29.00 | 1798.00 |
| 293A | genomic DNA | 62 | 1 | 20.00 | 20.00 | 1240.00 |
| 286 | genomic DNA | 62 | 1 | 9.24 | 9.24 | 572.88 |
| 280A | genomic DNA | 62 | 1 | 33.40 | 33.40 | 2070.80 |
| 312A | genomic DNA | 62 | 1 | 3.64 | 3.64 | 225.68 |
| 303C | genomic DNA | 62 | 1 | 15.60 | 15.60 | 967.20 |


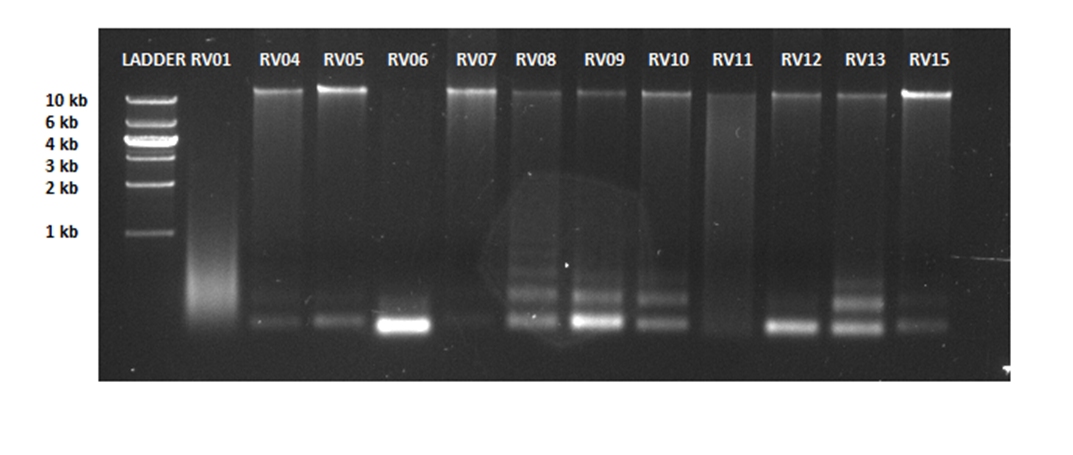
**Figure S1**. **Qualitative analysis of DNA from blood and tissue samples using gel electrophoresis.** Gel electrophoresis results for blood samples and pancreatic cancer donor (#250) tissue samples. Low yield DNA was not examined using gel electrophoresis to save adequate DNA for library preparation and

**Table S2. Sample Sequencing Statistics for whole exome sequencing** Overall sequencing quality and yield was measured for sequencing runs from cadaveric DNA pre-embalmed (blood) and embalmed samples ( donor 250 & 292).

| **Sample ID** | **# Reads** | **Yield (Mbases)** | **Mean Quality Score** | **% Bases >= 30** |
| --- | --- | --- | --- | --- |
| 2SM-292 | 75,571,463 | 22,671 | 38.88 | 93.97 |
| 7SC-292 | 74,171,309 | 22,251 | 38.88 | 93.98 |
| 6N-292 | 77,187,124 | 23,156 | 38.81 | 93.75 |
| 5L-292 | 68,240,142 | 20,472 | 38.8 | 93.66 |
| 3GM-292 | 79,175,921 | 23,753 | 38.75 | 93.49 |
| 1SM-292 | 79,576,733 | 23,873 | 38.65 | 93.1 |
| 286 | 38,280,255 | 11,484 | 37.31 | 92.79 |
| 306A | 26,069,032 | 7,821 | 35.79 | 93.12 |
| 303C | 21,078,886 | 6,324 | 35.46 | 91.29 |
| 275A | 26,382,958 | 7,915 | 35.59 | 92.11 |
| 272A | 21,331,090 | 6,399 | 35.79 | 93.16 |
| 281A | 19,622,210 | 5,887 | 35.87 | 93.52 |
| 293A | 35,734,468 | 10,720 | 37.07 | 92.6 |
| Kidney-250 | 23,502,410 | 7,051 | 35.45 | 91.59 |
| 311A | 27,630,757 | 8,289 | 35.84 | 93.4 |
| 312A | 36,655,654 | 10,997 | 37.47 | 93.08 |
| 280A | 24,206,046 | 7,262 | 35.84 | 93.4 |
| Pancreas-250 | 22,869,685 | 6,861 | 35.4 | 91.37 |
| 284A | 37,133,032 | 11,140 | 37.29 | 93.3 |
| Liver-250 | 35,436,070 | 10,631 | 35.27 | 90.83 |
| 298A | 19,403,943 | 5,821 | 35.82 | 93.31 |

**Table S3. Pathogenic variants reported in genomic reports. (A)** Donor ID, nearest gene, chromosomal location, variant consequence, and existing variation (SNP ID) for each pathogenic variant is included in this table. **(B)** Somatic genetic variation between 6 tissues in same donor (Prosection 292). Tissue sample naming- 1SM: Sartorius Muscle; 2SM =Striated Muscle; 3GM=Gluteus Maximus; 5L=Liver; 6N=Nerve; 7SC= Spinal cord nerve

**A.**

| **Donor** | **Variant Gene** | **Chromosome Location** | **Allele** | **Consequence** | **Existing_variation** |
| --- | --- | --- | --- | --- | --- |
| 272 | WARS2 | 1:119140608 | C | missense_variant | rs139548132 |
| 272 | MBL2 | 10:52771475 | T | missense_variant | rs1800450 |
| 272 | SAA1 | 11:18269312 | T | missense_variant | rs1136743 |
| 272 | APOA4 | 11:116820918 | A | missense_variant | rs5110 |
| 272 | TBX6 | 16:30091839 | A | upstream_gene_variant | rs3809627 |
| 272 | MAG | 19:35295965 | T | synonymous_variant | rs2301600 |
| 272 | ANO10 | 3:43605757 | - | frameshift_variant | rs758937084 |
| 272 | KLKB1 | 4:186236880 | A | missense_variant | rs3733402 |
| 272 | PLG | 6:160706469 | G | intron_variant,non_coding_transcript_variant | rs73015965 |
| 272 | GATA4 | 8:11748855 | C | intron_variant | rs10503425 |
| 272 | GATA4 | 8:11755333 | A | intron_variant | rs3729851 |
| 272 | GATA4 | 8:11758186 | G | intron_variant | rs745379 |
| 272 | GATA4 | 8:11759327 | A | 3_prime_UTR_variant | rs804290 |
| 275 | GNRHR2 | 1:145927447 | T | downstream_gene_variant | rs139428292 |
| 275 | MBL2 | 10:52771482 | A | missense_variant | rs5030737 |
| 275 | SAA1 | 11:18269312 | T | missense_variant | rs1136743 |
| 275 | MPO | 17:58278036 | A | missense_variant | rs28730837 |
| 275 | MAG | 19:35295965 | T | synonymous_variant | rs2301600 |
| 275 | FGFR4 | 5:177093242 | A | missense_variant | rs351855 |
| 275 | GATA4 | 8:11748803 | C | intron_variant | rs3735819 |
| 280 | MBL2 | 10:52771475 | T | missense_variant | rs1800450 |
| 280 | HBB | 11:5226925 | A | splice_region_variant,intron_variant | rs33915217 |
| 280 | SAA1 | 11:18269312 | T | missense_variant | rs1136743 |
| 280 | MAG | 19:35295965 | T | synonymous_variant | rs2301600 |
| 280 | CST3 | 20:23637790 | T | missense_variant | rs1064039 |
| 280 | WFS1 | 4:6293966 | G | intron_variant | rs6446482 |
| 280 | KLKB1 | 4:186236880 | A | missense_variant | rs3733402 |
| 280 | GATA4 | 8:11748803 | C | intron_variant | rs3735819 |
| 280 | GATA4 | 8:11755333 | A | intron_variant | rs3729851 |
| 280 | GATA4 | 8:11757260 | T | intron_variant | rs12156163 |
| 280 | GATA4 | 8:11759327 | A | 3_prime_UTR_variant | rs804290 |
| 280 | C1GALT1C1 | X:120626774 | T | missense_variant | rs17261572 |
| 281 | MBL2 | 10:52771482 | A | missense_variant | rs5030737 |
| 281 | STOX1 | 10:68885620 | C | missense_variant | rs10509305 |
| 281 | SAA1 | 11:18269312 | T | missense_variant | rs1136743 |
| 281 | PKD1 | 16:2106877 | A | synonymous_variant | rs752114168 |
| 281 | TBX6 | 16:30091839 | A | upstream_gene_variant | rs3809627 |
| 281 | ITGB4 | 17:75751112 | A | splice_donor_variant | rs147222357 |
| 281 | MAG | 19:35295965 | T | synonymous_variant | rs2301600 |
| 281 | KLKB1 | 4:186236880 | A | missense_variant | rs3733402 |
| 281 | GATA4 | 8:11748803 | C | intron_variant | rs3735819 |
| 281 | NDUFAF6 | 8:95035578-95035579 | A | splice_region_variant,intron_variant | rs34960210 |
| 284 | EPHB2 | chr1:22906853 | A | missense_variant | rs28936395 |
| 284 | GNRHR2 | chr1:145927447 | T | downstream_gene_variant | rs139428292 |
| 284 | SAA1 | chr11:18269312 | T | missense_variant | rs1136743 |
| 284 | PTPRJ | chr11:48123823 | C | missense_variant | rs1566734 |
| 284 | CRYGB | chr2:208146054 | - | intron_variant | rs3214759 |
| 284 | CYP27A1 | chr2:218814154 | T | missense_variant | rs41272687 |
| 284 | CST3 | chr20:23637790 | T | missense_variant | rs1064039 |
| 284 | BTD | chr3:15645186 | C | missense_variant | rs13078881 |
| 284 | KLKB1 | chr4:186236880 | A | missense_variant | rs3733402 |
| 284 | GATA4 | chr8:11748803 | C | intron_variant | rs3735819 |
| 284 | GATA4 | chr8:11758186 | G | intron_variant | rs745379 |
| 284 | GATA4 | chr8:11759327 | A | 3_prime_UTR_variant | rs804290 |
| 284 | NDUFAF6 | chr8:95035578-95035579 | A | splice_region_variant,intron_variant | rs34960210 |
| 286 | WARS2 | chr1:119140608 | C | missense_variant | rs139548132 |
| 286 | SAA1 | chr11:18269312 | T | missense_variant | rs1136743 |
| 286 | PTPRJ | chr11:48123823 | C | missense_variant | rs1566734 |
| 286 | TBX6 | chr16:30091481 | C | upstream_gene_variant | rs3809624 |
| 286 | TBX6 | chr16:30091839 | A | upstream_gene_variant | rs3809627 |
| 286 | CST3 | chr20:23637790 | T | missense_variant | rs1064039 |
| 286 | WFS1 | chr4:6293966 | G | intron_variant | rs6446482 |
| 286 | TGFBI | chr5:136062674 | C | missense_variant | rs121909217 |
| 286 | GATA4 | chr8:11748803 | C | intron_variant | rs3735819 |
| 286 | MT-ND3 | chrM:8839 | A | upstream_gene_variant | rs369202065 |
| 286 | OTC | chrX:38408967 | G | missense_variant | rs1800328 |
| 292 | STOX1 | 10:68885620 | C | missense_variant | rs10509305 |
| 292 | SAA1 | 11:18269312 | T | missense_variant | rs1136743 |
| 292 | NIN | 14:50744304 | C | missense_variant | rs387907308 |
| 292 | CP | 3:149182004 | G | splice_donor_variant | rs386134140 |
| 292 | KLKB1 | 4:186236880 | A | missense_variant | rs3733402 |
| 292 | IRGM | 5:150848436 | T | upstream_gene_variant | rs10065172 |
| 292 | GATA4 | 8:11748803 | C | intron_variant | rs3735819 |
| 292 | NDUFAF6 | 8:95035578-95035579 | A | splice_region_variant,intron_variant | rs34960210 |
| 292 | STOX1 | 10:68885620 | C | missense_variant | rs10509305 |
| 292 | SAA1 | 11:18269312 | T | missense_variant | rs1136743 |
| 292 | NIN | 14:50744304 | C | missense_variant | rs387907308 |
| 292 | FAM161A | 2:61840001 | T | 3_prime_UTR_variant,NMD_transcript_variant | rs777678022 |
| 292 | KLKB1 | 4:186236880 | A | missense_variant | rs3733402 |
| 292 | IRGM | 5:150848436 | T | upstream_gene_variant | rs10065172 |
| 292 | GATA4 | 8:11748803 | C | intron_variant | rs3735819 |
| 292 | NDUFAF6 | 8:95035578-95035579 | A | splice_region_variant,intron_variant | rs34960210 |
| 292 | SAA1 | 11:18269312 | T | missense_variant | rs1136743 |
| 292 | NIN | 14:50744304 | C | missense_variant | rs387907308 |
| 292 | BRIP1 | 17:61799235-61799236 | TAGCTGGGATTGCAGGCACA | frameshift_variant | rs730881647 |
| 292 | MLH1 | 3:37008863-37008864 | CCT | inframe_insertion | rs63749959 |
| 292 | NFKB1 | 4:102580641 | G | splice_donor_variant | rs869320689 |
| 292 | KLKB1 | 4:186236880 | A | missense_variant | rs3733402 |
| 292 | IRGM | 5:150848436 | T | upstream_gene_variant | rs10065172 |
| 292 | NSD1 | 5:177211226 | C | missense_variant | rs372407111 |
| 292 | GATA4 | 8:11748803 | C | intron_variant | rs3735819 |
| 292 | STOX1 | 10:68885620 | C | missense_variant | rs10509305 |
| 292 | SAA1 | 11:18269312 | T | missense_variant | rs1136743 |
| 292 | NALCN | 13:101292058 | T | missense_variant | rs878853133 |
| 292 | BRCA1 | 17:43092872-43092873 | CAA | intron_variant | rs80357541 |
| 292 | AC067945.1 | 2:190995184 | A | upstream_gene_variant | rs387906760 |
| 292 | KLKB1 | 4:186236880 | A | missense_variant | rs3733402 |
| 292 | IRGM | 5:150848436 | T | upstream_gene_variant | rs10065172 |
| 292 | GATA4 | 8:11748803 | C | intron_variant | rs3735819 |
| 292 | NDUFAF6 | 8:95035578-95035579 | AA | splice_region_variant,intron_variant | rs34960210 |
| 292 | MPDZ | 9:13119579 | C | non_coding_transcript_exon_variant | rs886043499 |
| 292 | CHM | X:85894214 | T | stop_gained | rs132630264 |
| 292 | STOX1 | 10:68885620 | C | missense_variant | rs10509305 |
| 292 | SAA1 | 11:18269312 | T | missense_variant | rs1136743 |
| 292 | NIN | 14:50744304 | C | missense_variant | rs387907308 |
| 292 | WFS1 | 4:6293966 | G | intron_variant | rs6446482 |
| 292 | KLKB1 | 4:186236880 | A | missense_variant | rs3733402 |
| 292 | IRGM | 5:150848436 | T | upstream_gene_variant | rs10065172 |
| 292 | GATA4 | 8:11748803 | C | intron_variant | rs3735819 |
| 292 | CRH | 8:66178947 | T | upstream_gene_variant | rs12721510 |
| 292 | NDUFAF6 | 8:95035578-95035579 | A | splice_region_variant,intron_variant | rs34960210 |
| 292 | STOX1 | 10:68885620 | C | missense_variant | rs10509305 |
| 292 | SAA1 | 11:18269312 | T | missense_variant | rs1136743 |
| 292 | NIN | 14:50744304 | C | missense_variant | rs387907308 |
| 292 | WFS1 | 4:6293966 | G | intron_variant | rs6446482 |
| 292 | KLKB1 | 4:186236880 | A | missense_variant | rs3733402 |
| 292 | IRGM | 5:150848436 | T | upstream_gene_variant | rs10065172 |
| 292 | GATA4 | 8:11748803 | C | intron_variant | rs3735819 |
| 292 | NDUFAF6 | 8:95035578-95035579 | A | splice_region_variant,intron_variant | rs34960210 |
| 293 | MIR181A1HG | chr1:198826991 | T | intron_variant,non_coding_transcript_variant | rs12406470 |
| 293 | SAA1 | chr11:18269312 | T | missense_variant | rs1136743 |
| 293 | CST3 | chr20:23637790 | T | missense_variant | rs1064039 |
| 293 | GATA4 | chr8:11748803 | C | intron_variant | rs3735819 |
| 293 | GATA4 | chr8:11758186 | G | intron_variant | rs745379 |
| 293 | NDUFAF6 | chr8:95035578-95035579 | A | splice_region_variant,intron_variant | rs34960210 |
| 293 | OTC | chrX:38408967 | G | missense_variant | rs1800328 |
| 298 | STOX1 | chr10:68885620 | C | missense_variant | rs10509305 |
| 298 | SAA1 | chr11:18269312 | T | missense_variant | rs1136743 |
| 298 | PTPRJ | chr11:48123823 | C | missense_variant | rs1566734 |
| 298 | PRSS53 | chr16:31091334 | A | upstream_gene_variant | rs72547528 |
| 298 | CYP27A1 | chr2:218814154 | T | missense_variant | rs41272687 |
| 298 | CHRND | chr2:232526603 | T | missense_variant | rs55868108 |
| 298 | KLKB1 | chr4:186236880 | A | missense_variant | rs3733402 |
| 298 | GATA4 | chr8:11758186 | G | intron_variant | rs745379 |
| 298 | PRDM12 | chr9:130681606-130681614 | - | inframe_deletion | rs752427775 |
| 303 | STOX1 | chr10:68885620 | C | missense_variant | rs10509305 |
| 303 | SAA1 | chr11:18269312 | T | missense_variant | rs1136743 |
| 303 | PTPRJ | chr11:48123823 | C | missense_variant | rs1566734 |
| 303 | TBX6 | chr16:30091481 | C | upstream_gene_variant | rs3809624 |
| 303 | CHST6 | chr16:75479230 | C | missense_variant | rs28937879 |
| 303 | CST3 | chr20:23637790 | T | missense_variant | rs1064039 |
| 303 | GATA4 | chr8:11748803 | C | intron_variant | rs3735819 |
| 303 | NDUFAF6 | chr8:95035578-95035579 | A | splice_region_variant,intron_variant | rs34960210 |
| 306 | STOX1 | chr10:68885620 | C | missense_variant | rs10509305 |
| 306 | SAA1 | chr11:18269312 | T | missense_variant | rs1136743 |
| 306 | PTPRJ | chr11:48123823 | C | missense_variant | rs1566734 |
| 306 | CRYGB | chr2:208146054 | - | intron_variant | rs3214759 |
| 306 | KLKB1 | chr4:186236880 | A | missense_variant | rs3733402 |
| 306 | FGFR4 | chr5:177093242 | A | missense_variant | rs351855 |
| 306 | PKHD1 | chr6:51659489 | - | frameshift_variant | rs770461067 |
| 306 | GATA4 | chr8:11748803 | C | intron_variant | rs3735819 |
| 306 | GATA4 | chr8:11757260 | T | intron_variant | rs12156163 |
| 306 | GATA4 | chr8:11758186 | G | intron_variant | rs745379 |
| 306 | GATA4 | chr8:11759327 | A | 3_prime_UTR_variant | rs804290 |
| 306 | NDUFAF6 | chr8:95035578-95035579 | A | splice_region_variant,intron_variant | rs34960210 |
| 306 | C1GALT1C1 | chrX:120626774 | T | missense_variant | rs17261572 |
| 311 | RHD | chr1:25303329 | G | missense_variant | rs121912763 |
| 311 | FMO3 | chr1:171107811 | T | missense_variant | rs72549326 |
| 311 | SAA1 | chr11:18269312 | T | missense_variant | rs1136743 |
| 311 | F2 | chr11:46725897 | A | missense_variant | rs62623459 |
| 311 | APOA4 | chr11:116820918 | A | missense_variant | rs5110 |
| 311 | CYP4F22 | chr19:15525513 | T | synonymous_variant | rs118091316 |
| 311 | CRYGB | chr2:208146054 | - | intron_variant | rs3214759 |
| 311 | WFS1 | chr4:6293966 | G | intron_variant | rs6446482 |
| 311 | KLKB1 | chr4:186236880 | A | missense_variant | rs3733402 |
| 311 | GATA4 | chr8:11748803 | C | intron_variant | rs3735819 |
| 311 | GATA4 | chr8:11758186 | G | intron_variant | rs745379 |
| 311 | MYMK | chr9:133519002 | T | missense_variant | rs137868995 |
| 311 | MT-ND4 | chrM:15812 | A | downstream_gene_variant | rs200336777 |
| 311 | SERPINA7 | chrX:106034370 | A | missense_variant | rs1804495 |
| 312 | MBL2 | chr10:52771482 | A | missense_variant | rs5030737 |
| 312 | STOX1 | chr10:68885620 | C | missense_variant | rs10509305 |
| 312 | SAA1 | chr11:18269312 | T | missense_variant | rs1136743 |
| 312 | CYP27A1 | chr2:218814154 | T | missense_variant | rs41272687 |
| 312 | NAGA | chr22:42061052 | T | missense_variant | rs121434529 |
| 312 | WFS1 | chr4:6293966 | G | intron_variant | rs6446482 |
| 312 | KLKB1 | chr4:186236880 | A | missense_variant | rs3733402 |
| 312 | PRLR | chr5:35072610 | G | missense_variant | rs72478580 |
| 312 | FGFR4 | chr5:177093242 | A | missense_variant | rs351855 |
| 312 | GATA4 | chr8:11748803 | C | intron_variant | rs3735819 |
| 312 | GATA4 | chr8:11758186 | G | intron_variant | rs745379 |
| 312 | NDUFAF6 | chr8:95035578-95035579 | A | splice_region_variant,intron_variant | rs34960210 |
| 312 | ABCD1 | chrX:153743056 | A | missense_variant | rs11146842 |

**B.**

| **Prosection** | **Sample** | **Variant Gene** |
| --- | --- | --- |
| 292 | 1_SM | STOX1 |
| 292 | 1_SM | SAA1 |
| 292 | 1_SM | NIN |
| 292 | 1_SM | CP |
| 292 | 1_SM | KLKB1 |
| 292 | 1_SM | IRGM |
| 292 | 1_SM | GATA4 |
| 292 | 1_SM | NDUFAF6 |
| 292 | 2_SM | STOX1 |
| 292 | 2_SM | SAA1 |
| 292 | 2_SM | NIN |
| 292 | 2_SM | FAM161A |
| 292 | 2_SM | KLKB1 |
| 292 | 2_SM | IRGM |
| 292 | 2_SM | GATA4 |
| 292 | 2_SM | NDUFAF6 |
| 292 | 3_GM | SAA1 |
| 292 | 3_GM | NIN |
| 292 | 3_GM | BRIP1 |
| 292 | 3_GM | MLH1 |
| 292 | 3_GM | NFKB1 |
| 292 | 3_GM | KLKB1 |
| 292 | 3_GM | IRGM |
| 292 | 3_GM | NSD1 |
| 292 | 3_GM | GATA4 |
| 292 | 5_L | STOX1 |
| 292 | 5_L | SAA1 |
| 292 | 5_L | NALCN |
| 292 | 5_L | BRCA1 |
| 292 | 5_L | AC067945.1 |
| 292 | 5_L | KLKB1 |
| 292 | 5_L | IRGM |
| 292 | 5_L | GATA4 |
| 292 | 5_L | NDUFAF6 |
| 292 | 5_L | MPDZ |
| 292 | 5_L | CHM |
| 292 | 6_N | STOX1 |
| 292 | 6_N | SAA1 |
| 292 | 6_N | NIN |
| 292 | 6_N | WFS1 |
| 292 | 6_N | KLKB1 |
| 292 | 6_N | IRGM |
| 292 | 6_N | GATA4 |
| 292 | 6_N | CRH |
| 292 | 6_N | NDUFAF6 |
| 292 | 7_SC | STOX1 |
| 292 | 7_SC | SAA1 |
| 292 | 7_SC | NIN |
| 292 | 7_SC | WFS1 |
| 292 | 7_SC | KLKB1 |
| 292 | 7_SC | IRGM |
| 292 | 7_SC | GATA4 |
| 292 | 7_SC | NDUFAF6 |

**Table S4. Overview of clinically relevant markers in pancreatic cancer and liver metastasis highlighting drug response variants. A.** An overview of all clinically relevant variants discovered between the pancreatic cancer tissue sample and liver metastases. **B**. Discovered drug response variants, which affect chemotherapeutic response. **C.** Pathogenic variants of primary pancreas and liver metastasis (italicized).

**A**.

| **Pancreas Primary Tumor Variants** | |
| --- | --- |
| Pathogenic | 11 |
| Likely Pathogenic | 4 |
| Drug Response | 8 |
| Uncertain Signficance | 156 |
| Risk Factor | 15 |
| Protective | 1 |
|  |  |
| **Liver Metastasis Variants** | |
| Pathogenic | 41 |
| Likely Pathogenic | 26 |
| Drug Response | 13 |
| Uncertain Significance | 286 |
| Risk Factor | 24 |
| Protective | 4 |

**B.**

| **SNP ID** | **Chromosome** | **Position** | **Overlapped Gene** | **Phenotypes** |
| --- | --- | --- | --- | --- |
| rs1751034 | 13 | 95062722 | ABCC4 | tenofovir response - |
| rs1532624 | 16 | 56971567 | CETP | HMG CoA reductase inhibitors response - Efficacy |
| rs2232228 | 16 | 69109674 | HAS3 | anthracyclines and related substances response - Toxicity/ADR |
| rs12943590 | 17 | 19716685 | SLC47A2 | metformin response - Efficacy |
| rs25487 | 19 | 43551574 | XRCC1 | carboplatin response - Efficacy, Toxicity/ADR, cisplatin response |
| rs3212986 | 19 | 45409478 | CD3EAP | cisplatin response - Toxicity/ADR, Platinum compounds response |
| rs3212986 | 19 | 45409478 | ERCC1 | cisplatin response - Toxicity/ADR, Platinum compounds response |
| rs7270101 | 20 | 3213247 | ITPA | Inosine triphosphatase deficiency, peginterferon alfa-2b and ribavirin response |
| rs1056892 | 21 | 36146408 | CBR3-AS1 | anthracyclines and related substances response - Toxicity/ADR |
| rs1056892 | 21 | 36146408 | CBR3 | anthracyclines and related substances response - Toxicity/ADR |
| rs1042713 | 5 | 148826877 | ADRB2 | Asthma, nocturnal, susceptibility to, Metabolic syndrome, susceptibility to |
| rs20455 | 6 | 39357302 | KIF6 | atorvastatin response - Efficacy, pravastatin response - Efficacy |
| rs1726866 | 7 | 141972905 | MGAM | Phenylthiocarbamide tasting |
| rs1726866 | 7 | 141972905 | TAS2R38 | Phenylthiocarbamide tasting |
| rs1041983 | 8 | 18400285 | NAT2 | ethambutol, isoniazid, pyrazinamide, and rifampin response - Toxicity/ADR |
| rs1799930 | 8 | 18400593 | NAT2 | Slow acetylator due to N-acetyltransferase enzyme variant,ethambutol, and rifampin response |

**C**.

| **Sample** | **SNP ID** | **Overlapped Gene** | **Phenotypes** |
| --- | --- | --- | --- |
| Pancreas-Primary | rs1800450 | MBL2 | Mannose-binding protein deficiency |
| Pancreas-Primary | rs80358998 | BRCA2 | Hereditary cancer-predisposing syndrome,Hereditary breast & ovarian ccr |
| Pancreas-Primary | rs398122999 | CHD2 | Epileptic encephalopathy, childhood-onset |
| Pancreas-Primary | rs1805010 | IL4R | Acquired immunodeficiency syndrome, slow progression to, Atopy |
| Pancreas-Primary | rs2285892 | NF1 | Neurofibromatosis, type 1 |
| Pancreas-Primary | rs799917 | BRCA1 | Familial cancer of breast,Hereditary breast and ovarian cancer syndrome |
| Pancreas-Primary | rs397508986 | BRCA1 | Familial cancer of breast,Hereditary breast and ovarian cancer syndrome |
| Pancreas-Primary | rs2301600 | MAG | Spastic paraplegia 75, autosomal recessive |
| Pancreas-Primary | rs429358 | APOE | Alzheimer disease 2,not provided |
| Pancreas-Primary | rs2814778 | CADM3-AS1 | DUFFY BLOOD GROUP SYSTEM, FY(a-b-) PHENOTYPE,Plasmodium vivax |
| *Liver-Metasasis* | *chr10:95613755:A/G:1* | *ALDH18A1* | *Cutis laxa-corneal clouding-oligophrenia syndrome;Spastic paraplegia 9b, autosomal recessive* |
| *Liver-Metasasis* | *chr11:44114224:C/G:1* | *EXT2* | *Multiple exostoses type 2* |
| *Liver-Metasasis* | *chr12:6869369:G/T:1* | *TPI1* | *Triosephosphate isomerase deficiency* |
| *Liver-Metasasis* | *chr13:110187283:C/T:1* | *COL4A1* | *Angiopathy, hereditary, with nephropathy, aneurysms, and muscle cramps* |
| *Liver-Metasasis* | *chr13:32325169:C/-:1* | *BRCA2* | *Familial cancer of breast,Breast-ovarian cancer, familial 2* |
| *Liver-Metasasis* | *chr13:32332779:AAAG/-:1* | *BRCA2* | *Hereditary cancer-predisposing syndrome,Hereditary breast and ovarian cancer syndrome* |
| *Liver-Metasasis* | *chr13:32337751:A/-:1* | *BRCA2* | *Fanconi anemia,Hereditary cancer-predisposing syndrome,Familial cancer of breast,* |
| *Liver-Metasasis* | *chr16:27344882:A/C/G/T:1* | *IL4R* | *Acquired immunodeficiency syndrome, slow progression to,Atopy, resistance to* |
| *Liver-Metasasis* | *chr17:19343795:C/T:1* | *B9D1* | *Joubert syndrome 27* |
| *Liver-Metasasis* | *chr17:31226467:G/A:1* | *NF1* | *Hereditary cancer-predisposing ,Neurofibromatosis, type 1,Café-au-lait /pulmonary stenosis* |
| *Liver-Metasasis* | *chr17:43071077:T/-:1* | *BRCA1* | *Hereditary cancer-predisposing syndrome,Hereditary breast and ovarian cancer syndrome* |
| *Liver-Metasasis* | *chr17:43091852:G/A:1* | *BRCA1* | *Familial cancer of breast,Breast-ovarian cancer, familial 1* |
| *Liver-Metasasis* | *chr17:43092919:G/A/C/T:1* | *BRCA1* | *Familial cancer of breast,Hereditary breast and ovarian cancer syndrome,* |
| *Liver-Metasasis* | *chr17:43094404:T/-:1* | *BRCA1* | *Hereditary breast and ovarian cancer syndrome,Breast-ovarian cancer, familial 1* |
| *Liver-Metasasis* | *chr17:43124063:G/A:1* | *BRCA1* | *Familial cancer of breast,Hereditary breast and ovarian cancer syndrome* |
| *Liver-Metasasis* | *chr19:35295965:C/G/T:1* | *MAG* | *Spastic paraplegia 75, autosomal recessive* |
| *Liver-Metasasis* | *chr19:44908684:T/C:1* | *APOE* | *Alzheimer disease 2,not provided* |
| *Liver-Metasasis* | *chr19:46846102:C/A/T:1* | *AP2S1* | *Hypocalciuric hypercalcemia, familial, type III* |
| *Liver-Metasasis* | *chr1:159204893:T/C:1* | *CADM3-AS1* | *DUFFY BLOOD GROUP SYSTEM, FY(a-b-) PHENOTYPE,Plasmodium vivax, resistance to,* |
| *Liver-Metasasis* | *chr1:197104018:G/A:1* | *ASPM* | *Primary autosomal recessive microcephaly 5* |
| *Liver-Metasasis* | *chr1:225422087:G/A:1* | *LBR* | *Pelger-HuÃ«t anomaly* |
| *Liver-Metasasis* | *chr1:63631761:C/T:1* | *PGM1* | *Congenital disorder of glycosylation,Congenital disorder of glycosylation type 1t,not specified* |
| *Liver-Metasasis* | *chr1:99861676:C/T:1* | *AGL* | *Glycogen storage disease type III* |
| *Liver-Metasasis* | *chr2:190995205:G/A:1* | *STAT1* | *Immunodeficiency 31C* |
| *Liver-Metasasis* | *chr4:186236880:G/A/C:1* | *KLKB1* | *Prekallikrein deficiency* |
| *Liver-Metasasis* | *chr4:6293966:C/G:1* | *WFS1* | *Diabetes mellitus, noninsulin-dependent, association with* |
| *Liver-Metasasis* | *chr4:87612388:A/T:1* | *AC093895.1* | *Dentinogenesis imperfecta - Shield's type II,not specified* |
| *Liver-Metasasis* | *chr5:142314268:C/T:1* | *SPRY4* | *Hypogonadotropic hypogonadism 17 with or without anosmia* |
| *Liver-Metasasis* | *chr5:151343594:C/A:1* | *SLC36A2* | *Hyperglycinuria,Iminoglycinuria, digenic* |
| *Liver-Metasasis* | *chr5:78129204:A/T:1* | *AP3B1* | *Hermansky-Pudlak syndrome,not specified* |
| *Liver-Metasasis* | *chr7:5997349:G/-:1* | *PMS2* | *Hereditary cancer-predisposing syndrome,Lynch syndrome,not specified* |
| *Liver-Metasasis* | *chr8:125055601:C/G:1* | *WASHC5* | *Spastic paraplegia 8* |
| *Liver-Metasasis* | *chr8:41797646:A/G:1* | *ANK1* | *Spherocytosis, type 1, autosomal recessive* |
| *Liver-Metasasis* | *chr9:94603438:T/CC:1* | *FBP1* | *Fructose-biphosphatase deficiency,not specified* |
| *Liver-Metasasis* | *chrX:54014529:G/A:1* | *PHF8* | *Siderius X-linked mental retardation syndrome* |
